# Frequency dependence of CA3 spike phase response arising from h-current properties

**DOI:** 10.3389/fncel.2013.00263

**Published:** 2013-12-25

**Authors:** Melodie Borel, Simone Guadagna, Hyun Jae Jang, Jeehyun Kwag, Ole Paulsen

**Affiliations:** ^1^Department of Physiology, Development and Neuroscience, University of CambridgeCambridge, UK; ^2^Department of Brain and Cognitive Engineering, Korea UniversitySeoul, Korea

**Keywords:** theta oscillation, phase response, *I*_*h*_, resonance, hippocampus, CA3, CA1

## Abstract

The phase of firing of hippocampal neurons during theta oscillations encodes spatial information. Moreover, the spike phase response to synaptic inputs in individual cells depends on the expression of the hyperpolarization-activated mixed cation current (*I*_*h*_), which differs between CA3 and CA1 pyramidal neurons. Here, we compared the phase response of these two cell types, as well as their intrinsic membrane properties. We found that both CA3 and CA1 pyramidal neurons show a voltage sag in response to negative current steps but that this voltage sag is significantly smaller in CA3 cells. Moreover, CA3 pyramidal neurons have less prominent resonance properties compared to CA1 pyramidal neurons. This is consistent with differential expression of *I*_*h*_ by the two cell types. Despite their distinct intrinsic membrane properties, both CA3 and CA1 pyramidal neurons displayed bidirectional spike phase control by excitatory conductance inputs during theta oscillations. In particular, excitatory inputs delivered at the descending phase of a dynamic clamp-induced membrane potential oscillation delayed the subsequent spike by nearly 50 mrad. The effect was shown to be mediated by *I*_*h*_ and was counteracted by increasing inhibitory conductance driving the membrane potential oscillation. Using our experimental data to feed a computational model, we showed that differences in *I*_*h*_ between CA3 and CA1 pyramidal neurons could predict frequency-dependent differences in phase response properties between these cell types. We confirmed experimentally such frequency-dependent spike phase control in CA3 neurons. Therefore, a decrease in theta frequency, which is observed in intact animals during novelty, might switch the CA3 spike phase response from unidirectional to bidirectional and thereby promote encoding of the new context.

## 1. Introduction

During spatial exploration, the rodent hippocampus exhibits a distinctive rhythmic slow network activity, during which the extracellularly recorded local field potential shows oscillations at theta frequency (4–12 Hz; Vanderwolf, 1969; O'Keefe and Recce, 1993; Buzsáki, 2002). During theta oscillations in anaesthetized rats, the membrane potential of hippocampal pyramidal neurons is driven by rhythmic perisomatic inhibition (Soltész and Deschenes, 1993; Kamondi et al., 1998). As a consequence, their spike probability distribution is phase locked to the ongoing theta rhythm (Kamondi et al., 1998). However, pyramidal neurons are not fully synchronized and the phase relative to theta oscillation at which each of them is active carries information, as shown by the phase precession of place cell firing when an animal moves through an environment (O'Keefe and Recce, 1993). The hyperpolarization-activated current, *I*_*h*_, mediated by HCN1 channels, confers to hippocampal neurons a frequency preference to inputs in the theta range (Hu et al., 2002). Nevertheless, theta power is increased in the hippocampus of animals with a genetic knockout of HCN1 channels (Nolan et al., 2004), suggesting that h-channels are more important for spike phase control in individual neurons, rather than contributing to the synchronization of neuronal firing during theta activity.

Several lines of experimental evidence suggest that hippocampal theta oscillations are important for memory processes: (i) Theta activity occurs during learning: the theta power increases during a water maze task when the rat has to learn the location of a hidden platform and not when it can see it (Olvera-Cortés et al., 2002). (ii) The power of theta oscillation correlates with performance: rabbits develop eyeblink conditioning twice as fast if stimuli are delivered when the hippocampus displays theta oscillations (Seager et al., 2002). Moreover, during food foraging on a hole-board, theta power is stronger when animals have the possibility of learning the position of baited/unbaited holes. This activity is associated with learning as demonstrated by a decrease in the rate of visiting unbaited holes (Woldeit and Korz, 2010). (iii) Theta activity is required for performance: suppression of rhythmic neuronal activity in rat hippocampus, resulting from a lesion (Winson, 1978) or pharmacological inactivation (McNaughton et al., 2006) of the medial septum, prevents them from learning the target position of a circular maze or water maze, respectively. (iv) Restoration of theta oscillations rescues performance: a rhythmic electrical stimulation of the septohippocampal fibres, while the medial septum is inactivated, partially restores theta-like activity in the rat hippocampus and considerably improves performance in the Morris water maze task (McNaughton et al., 2006). Finally, (v) theta activity also occurs during rapid eye movement (REM) sleep (Jouvet et al., 1959), which appears to be involved in memory consolidation: the duration of REM sleep increases specifically after a rat is trained in a water maze task (Smith and Rose, 1997), and place cells reactivate during REM episodes following exposure to the corresponding place field (Pavlides and Winson, 1989). A rat that is deprived of REM sleep for 12 hours after its first training session in a water maze task takes longer time to find the hidden platform in a second session (Smith and Rose, 1996). Interestingly, an enhancement of REM sleep following the training of rats in a footshock-motivated discrimination task improves their performance (Wetzel et al., 2003).

The mechanism by which theta oscillations contribute to memory is unknown but might involve plasticity of synaptic transmission. Huerta and Lisman (1993) reported that field excitatory post-synaptic potentials (EPSPs) recorded in the *stratum radiatum* of CA1 are potentiated in hippocampal slices when the Schaffer collateral pathway is stimulated at low frequency during carbachol-induced theta oscillations. In particular, the magnitude of the potentiation correlates with the amplitude of the oscillation (Huerta and Lisman, 1993). This long-lasting and activity-dependent strengthening of synaptic transmission also occurs in rats under anaesthesia during spontaneous or pinched-induced theta oscillation (Hölscher et al., 1997). Importantly, electrical stimulation of the afferent path induces long term potentiation only when it occurs at the positive phase of the field theta oscillation. Such a synaptic input, given the relationship between the field theta cycle and the spike distribution of pyramidal neurons (Kamondi et al., 1998), occurs slightly before pyramidal neurons are the most likely to fire an action potential. This form of synaptic plasticity has been reproduced at the single neuron level *in vitro* (Kwag and Paulsen, 2009b) and Schaffer collateral stimulation at the ascending phase of the membrane potential oscillation potentiates EPSPs when the post-synaptic cell fires at the peak of the oscillation. In contrast, synaptic stimulation at the descending phase of the membrane potential oscillation, after the action potential at the peak of the oscillation, depresses the EPSPs. Interestingly, a phase shift of the post-synaptic action potential influences the direction of the synaptic plasticity due to the time dependence between pre- and post-synaptic activity (Kwag and Paulsen, 2009b). Therefore, control of the post-synaptic spike phase could determine the direction and extent of synaptic plasticity.

Evidence of such spike phase control by synaptic inputs has previously been reported in rats in both CA3 (Lengyel et al., 2005) and CA1 pyramidal neurons (Kwag and Paulsen, 2009a). However, CA3 and CA1 differ considerably anatomically and are likely to have distinct functions in memory processes. While both CA3 and CA1 pyramidal neurons receive direct information from the entorhinal cortex, they also receive major inputs via the tri-synaptic circuit from the dentate gyrus to CA3 and then CA1 (Amaral and Witter, 1989). Because of its abundant recurrent connections, CA3 is thought to support autoassociative memory and to encode context (McNaughton and Morris, 1987). Rats with specific lesions of the CA3 subfield, although perfectly able to detect the novelty of an object they never have encountered before, fail to detect the spatial novelty if a familiar object is moved (Lee et al., 2005). The CA1, which receives the internally encoded input from CA3 and direct information from the entorhinal cortex, is thought to support heteroassociative memory of novelty (McNaughton and Morris, 1987). The quantification of c-fos protein expression in CA1 of rats exposed to an environment was shown to be positively correlated to the degree of novelty (VanElzakker et al., 2008). Interestingly, training of an animal in a navigation task induces a backward shift in place fields of hippocampal cells (Mehta et al., 1997). This asymmetric expansion of place fields is NMDAR-dependent (Ekstrom et al., 2001), and is reduced in HCN1 knockout mice (Hussaini et al., 2011). Moreover, CA3 and CA1 place cells have distinct properties with regard to this experience-dependent change of activity (Lee et al., 2004).

Here, we compared the intrinsic membrane properties of mouse CA3 and CA1 pyramidal neurons and analyzed the consequence of their differences on spike phase control by synaptic input.

## 2. Materials and methods

### 2.1. Preparation of the biological material

Animals used in this study were C57BL/6 mice (Harlan, Wyton, UK; *n* = 51) of both sexes, aged 2 to 5 weeks. To investigate the effect of the hyperpolarization-activated current *I*_*h*_, some experiments were carried out on mice with a genetic knockout of the hyperpolarization-activated cyclic nucleotide-gated channel 1 (HCN1 KO; B6.129S-Hcn1^tm2Kndl^/J, The Jackson Laboratory, Bar Harbor, Maine, USA; *n* = 3). All animal care and experimental procedures were in accordance with the UK Animals (Scientific Procedures) Act of 1986.

Horizontal hippocampal slices (350 μm) were prepared from the left hemisphere in cold (0–3 °*C*), oxygenated (95% O_2_, 5% CO_2_), sucrose-based cutting solution (in mM: KCl 3, NaH_2_PO_4_ 1.25, MgSO_4_ 2, MgCl_2_ 1, CaCl_2_ 1, NaHCO_3_ 26.4, glucose 10, sucrose 206, ascorbic acid 0.40, kynurenic acid 1) using a vibrating microtome (VT1200S, Leica Micro-systems, Milton Keynes, UK). Slices were stored at room temperature in a submerged-style holding chamber with oxygenated artificial cerebrospinal fluid (aCSF; in mM: NaCl 126, KCl 3, NaH_2_PO_4_ 1.25, MgSO_4_ 2, CaCl_2_ 2, NaHCO_3_ 26.4, glucose 10) for at least 1 h. Kynurenic acid (0.5 mM; Abcam, Cambridge, UK) was added to standard aCSF for the first 30 min of their recovery.

Slices were then individually placed in a recording chamber, superfused with oxygenated, standard aCSF at 30 °*C* at a flow-rate of approximately 3.5 mL·min^−1^. For some experiments, the *I*_*h*_ blocker ZD7288 (10 μM; Sigma Aldrich, Dorset, UK) was added to the superfusate 20 min prior to starting recordings.

### 2.2. Recording and stimulation

#### 2.2.1. Whole-cell recording

Whole-cell patch-clamp recordings of CA3 and CA1 pyramidal neurons were performed with a Multiclamp 700B amplifier (Molecular Devices, Foster City, California, USA) in current clamp mode under visual guidance by infrared differential interference contrast video microscopy. Patch electrodes (4–7 MΩ) were pulled from borosilicate glass capillaries and filled with a solution containing (in mM): K gluconate 110, HEPES 40, NaCl 4, ATP-Mg 4, GTP-NaCl 0.3; the pH was adjusted to 7.2 with KOH. Amplified signals were filtered at 4 kHz and digitized at 8 kHz (Instrutech ITC-18, Port Washington, New York, USA). Customized procedures within Igor Pro Software (WaveMetrics, Lake Oswego, Oregon, USA) were used to generate command signals, and for data collection and analysis.

#### 2.2.2. Dynamic clamp

Cells were recorded in dynamic clamp mode (Robinson and Kawai, 1993; Prinz et al., 2004) to allow stimulations mimicking physiological synaptic inputs. The injected current (*I*_inj_) was calculated as:

(1) $$I_{\text{inj}}(t)=g(t)\times [V_{m}(t)-E_{\text{Rev}}]$$

where *g*(*t*) is the conductance as a function of time, *V*_*m*_(*t*) is the measured membrane potential and *E*_Rev_ is the reversal potential of the synaptic input to be mimicked. The reversal potential was set to −70 mV for inhibitory inputs and to 0 mV for excitatory inputs.

Occasionally, the experiments required the membrane potential of the cell to be clamped using dynamic clamp mode. The reversal potential was then set to the clamp value and *g*(*t*) was set to be constant at its maximal value (5 nS).

### 2.3. Experimental design

Pyramidal neurons from the CA3 and CA1 subfields were identified on the basis of location, as well as morphological and electrical properties.

#### 2.3.1. Characterization of the cell

The cell resting membrane potential (RMP) was defined as the average potential recorded over 1250 ms without any injected current. Cells with RMP positive to −50 mV were discarded. The sag amplitude, reflecting the activation of *I*_*h*_, was measured at the soma from the membrane potential response to a negative current step (−100 pA, 800 ms), from a steady state membrane potential of −60 mV.

(2) $$\text{Sag}=\frac{V_{\text{peak}}-V_{\text{steady}}}{V_{\text{peak}}-\text{RMP}}$$

The resonance properties of the membrane were studied using an impedance (*Z*) amplitude profile (ZAP) protocol as previously described (Pike et al., 2000). An oscillatory current of constant peak-to-peak amplitude (40 pA) and increasing frequency (from 0 to 20 Hz) was delivered at the soma held close to −60, −70, or −80 mV by superimposing a constant current. Both the stimulation current command and the voltage response were subjected to a discrete Fourier transform (FFT). The ratio of the response FFT over the stimulation FFT determines the impedance of the cell (Puil et al., 1986). A resonance frequency was found when the impedance reached a frequency-specific peak (*Z*_res_). The strength of the resonance was calculated as the “*Q*-value” which was estimated as the ratio of *Z*_res_ over *Z*_0.5 Hz_ (Hu et al., 2002).

#### 2.3.2. Characterization of the spike phase control

An 11 s-long oscillatory current was delivered at the soma from a sinusoidal inhibitory conductance using dynamic clamp (5 Hz, either 3 or 1 nS). A tonic current was superimposed on the oscillatory input so that one action potential was triggered at the peak of each cycle of the oscillation. Five dynamic clamp-induced artificial excitatory post-synaptic conductances (aEPSGs) per trial were modeled using an alpha function:

(3) $$\text{aEPSG}(t)=g_{\text{max}}\times \alpha t\times e^{\left(1-\alpha t\right)}$$

with *g*_max_ = 1 nS and α = 260. They occurred at 20 different phases of the oscillation over 40 trials within the time intervals: [2,2.4], [4,4.4], [6,6.4], [8,8.4], and [10,10.4] s.

The four cycles immediately preceding each aEPSG were used as a control period from which to calculate the average spike phase, which was used as a reference. The phase of each aEPSG and that of the subsequent spike were compared to this reference. The phase responses were averaged in 20 bins of 0.1π rad. The effect of the aEPSG on the subsequent spike has been reported as phase responses in the text. In some figure panels the spike time response has been plotted as a function of input phase.

A related protocol was designed to study the phase response specifically to inputs at the descending phase of the membrane potential oscillation. In this protocol, the dynamic clamp-induced 5 nS oscillation at either 5 or 4 Hz lasted for 7 s. Each trial included a perturbation whose duration was set to half of the duration of the descending phase (between 0.3π and 0.8π rad). Sub-threshold excitation and inhibition were mimicked by clamping the membrane potential for this duration just negative to the spike threshold or at the trough of the oscillation, respectively. A third, control, condition measured the variation of spike phase in the absence of any induced perturbation. These three conditions were alternated, 15 trials of each were recorded and the response presented as the average phase response.

### 2.4. Statistical analysis

Conventional and circular statistics (Berens, 2009) were used as detailed in the Results section. As a test of normality, the D'Agostino–Pearson omnibus test (*p* > 0.05) was used prior to applying a Student's *t*-test. A circular one-sample mean angle test was applied to compare the spike phase distribution from a reference value. Differences between two independent spike phase distributions were tested using the Watson–Williams test. Comparison of spike phase shifts in several conditions per cell were tested using the Moore's paired test. A *p*-value < 0.05 was considered statistically significant.

### 2.5. Computational model

Hippocampal CA3 and CA1 pyramidal neurons were modeled as a single compartment neuron model using the NEURON program [Version 7.2, Hines and Carnevale, 1997]. CA3 and CA1 neurons were modeled to have the passive membrane properties as shown in Table 1 and CA3 neurons were modeled to have a larger surface area than CA1 neurons (Ishizuka et al., 1995; Morellini et al., 2010).

**Table 1:** **Morphology and passive membrane properties of CA3 and CA1 pyramidal cell (PC) models**.

		**CA3 PC**	**CA1 PC**
Soma	Length (μm)	75	60
	Diameter (μm)	60	60
Resting potential (mV)		−60	−60
Membrane capacitance (μF·cm^−2^)		1.0	1.0
Membrane resistance (kΩ·cm^2^)		200	100
Axial resistance (Ω·cm)		50	50

All voltage-gated conductances were modeled using Hodgkin-Huxley style kinetics (Hodgkin and Huxley, 1952). Leak (*I*_*Leak*_), fast sodium (*I*_*Na*_), delayed-rectifier potassium (*I*_*KDR*_) and A-type potassium (*I*_*KA*_) currents were modeled (Morse et al., 2010) in both the CA3 and CA1 models, each with a maximal conductance as shown in Table 2.

**Table 2:** **Maximal conductance of voltage-gated conductance in CA1 and CA3 pyramidal neuron model**.

	**Conductance (mS·cm^−2^)**
*g*_*Leak*_	0.01
*g*_*Na*_	9
*g*_*KDR*_	6
*g*_*KA*_	36

#### 2.5.1. Activation kinetics of CA3 and CA1 h-channels

**Figure 1:**
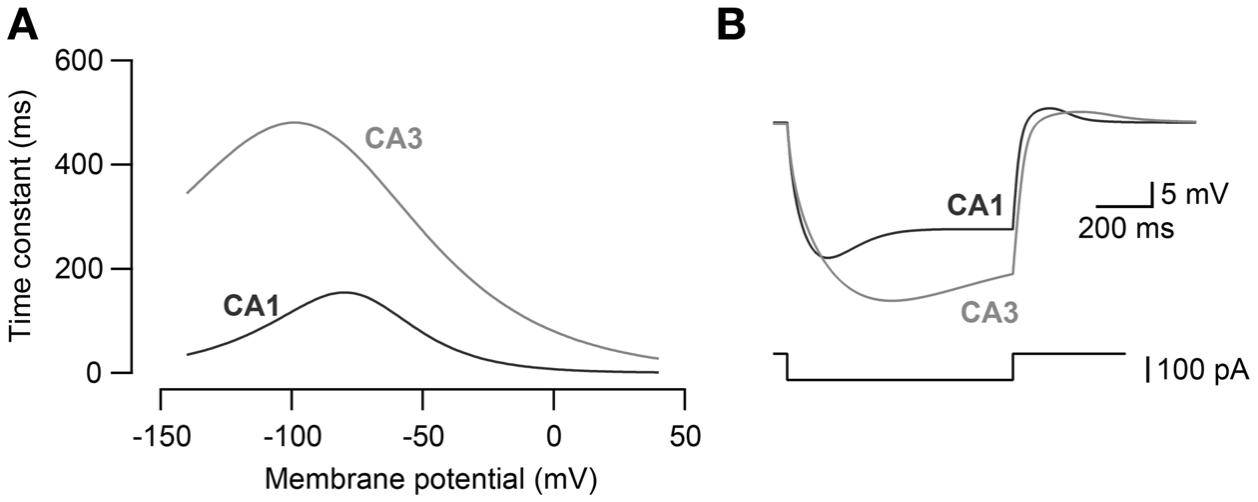
**Modeling of *I*_*h*_ kinetics. (A)** The activation time constants of h-channel conductance for CA3 pyramidal neuron model and CA1 pyramidal neuron model. **(B)** Voltage response to −100 pA current steps (bottom) in CA3 and CA1 neuron models. CA3 and CA1 neuron models were fit to the voltage response recorded in CA3 and CA1 pyramidal neurons to −100 pA current steps *in vitro*.

The kinetics of *I*_*h*_ in CA3 and CA1 models (Morse et al., 2010) were adjusted (Figure 1A) so that the voltage sag amplitude in response to steps of current reflected the experimental recordings of the CA3 and CA1 neurons *in vitro* (Figure 1B). The maximal h-channel conductance (*g*_*h*_) of CA3 was 2.0 × 10^−3^ mS·cm^−2^ and that of CA1 neuron was 4.5 × 10^−3^ mS·cm^−2^. The activation kinetics of h-channels for CA3 and CA1 pyramidal neuron models were adopted from Morse et al. (2010; Equations 4–5):

(4) $$\frac{dm}{dt}=\left[1-\left(\frac{1}{1+e^{-(V_{m}+73)/8}}-m\right)\right]\times \frac{1}{\tau_{\left\{CA1,CA3\right\}}}$$

where *m* was the gating variable, *V*_*m*_ was the membrane potential and τ was the time constant with:

(5) $$\tau_{\text{CA3}}=942\times e^{-(V_{m}\;+\;90)/37}$$

and

(6) $$\tau_{\text{CA1}}=303\times e^{-(V_{m}\;+\;75)/20}$$

Time constants were modified for CA3 (Equation 5) and CA1 neuron model (Equation 6) to fit the voltage response to hyperpolarizing current steps obtained experimentally in CA3 and CA1 neurons (Figure 1B).

#### 2.5.2. Simulation conditions and analysis

Oscillation of the membrane potential was simulated with an oscillatory inhibitory conductance at 5 Hz or 4 Hz (1 s, 4 nS), and a step current (1 s, 20.0 − 24.3 pA) was superimposed so that the model elicited one spike at the peak of each oscillation cycle. To simulate artificial synaptic input onto the cell, an excitatory or inhibitory conductance step (5 nS) was delivered on the descending phase of the oscillation (0.3 − 0.8π rad). The spike phase response to excitatory or inhibitory perturbation was calculated in the CA3 and CA1 models, compared to the spike phase before perturbation. All NEURON simulations were conducted with a time step of 25 μs and the total duration of a simulation was 1000 ms.

## 3. Results

### 3.1. Passive and resonance properties of CA3 and CA1 pyramidal neurons

**Figure 2:**
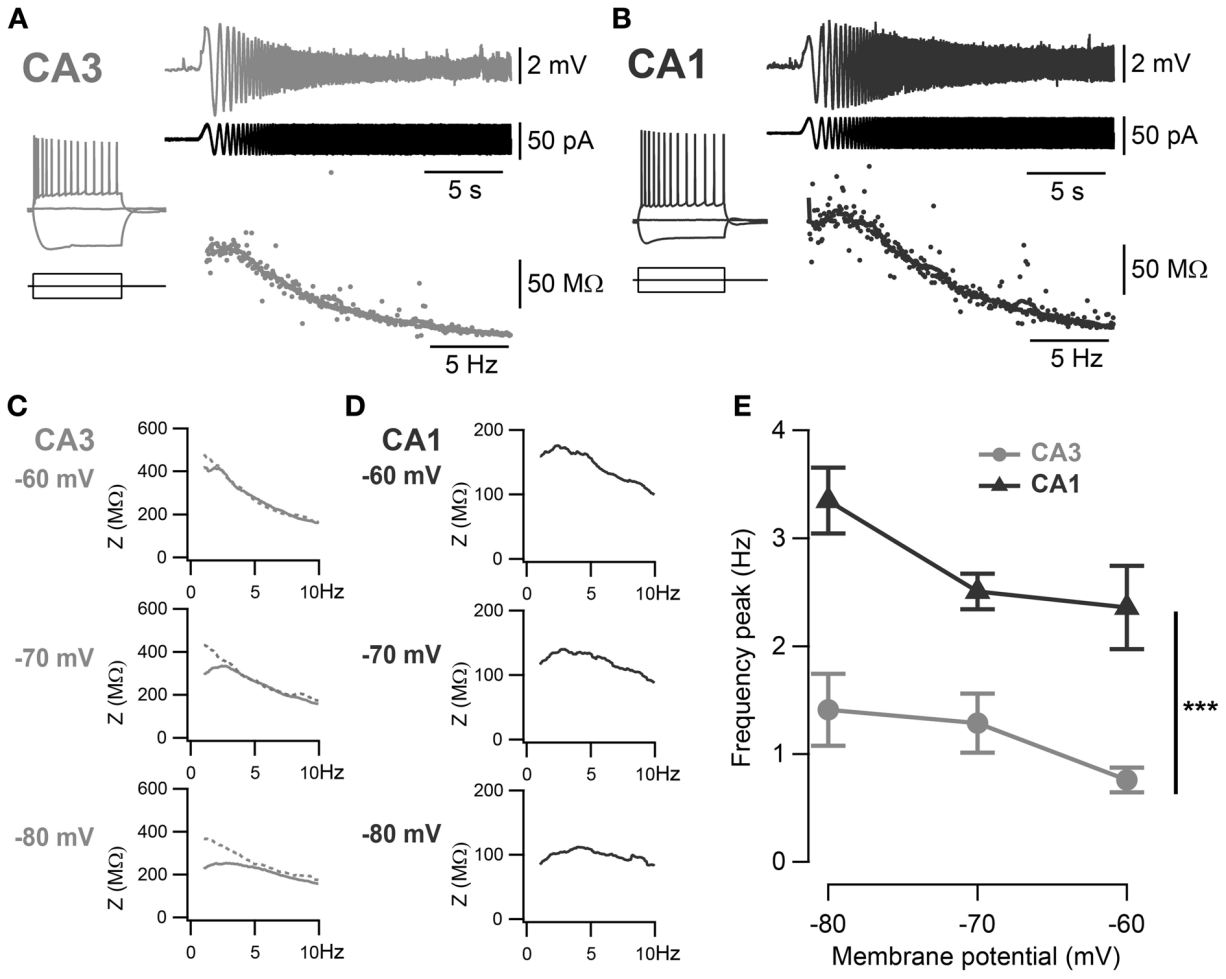
**Frequency preference of CA3 and CA1 pyramidal neurons. (A,B)** Representative ZAP recordings of CA3 and CA1 cells, respectively. Voltage responses (upper traces) to a sinusoidal current of increasing frequency (0–20 Hz, middle traces) were recorded and relative impedances (lower traces) were calculated. A peak in the impedance curve indicates a frequency preference of the cell. Representative impedance magnitudes of CA3 **(C)** and CA1 **(D)** pyramidal neurons at −60, −70 and −80 mV holding potentials. Some cells in CA3 did not show detectable frequency preference (dotted line) and a frequency preference could not be detected. **(E)** Average frequency preference of CA3 cells with resonance peak above 1 Hz (*n* = 7) and all CA1 cells (*n* = 12). Preferred frequency increased at more negative holding potentials and was significantly greater in CA1 than in CA3 cells (^***^p < 0.001, One-Way ANOVA with Bonferroni *post-hoc* test).

In order to compare the intrinsic membrane properties of CA1 and CA3 pyramidal neurons, whole-cell recordings were made in mouse hippocampal slices. The RMP of CA3 and CA1 pyramidal neurons (CA3: −54 ± 1 mV, *n* = 40; CA1: −57 ± 1 mV, *n* = 21; *p* = 0.06, unpaired two-sample Student's *t*-test; Table 3), as well as their input resistance (CA3: 248 ± 10 MΩ, *n* = 40; CA1: 212 ± 16 MΩ, *n* = 21; *p* = 0.07, unpaired two-sample Student's *t*-test; Table 3) were comparable. However, CA3 and CA1 neurons showed distinct sag amplitudes in response to negative current steps (see representative traces in Figures 2A,B). When recorded at −60 mV, the sag ratio in CA1 (0.30 ± 0.02, *n* = 21) was twice that in CA3 neurons (0.16 ± 0.01, *n* = 40; *p* < 10^−4^, unpaired two-sample Student's *t*-test; Table 3).

**Table 3:** **Passive membrane properties and resonance properties of CA3 and CA1 pyramidal neurons**.

	**CA3**	**CA1**	
RMP (mV)	−54 ± 1 (40)	−57 ± 1 (21)	n.s.
Rin (MΩ)	248 ± 10 (40)	212 ± 16 (21)	n.s.
Sag	0.16 ± 0.01 (40)	0.30 ± 0.02 (21)	***
F_Res_ (Hz)	1.19 ± 0.09 (15)	2.49 ± 0.17 (19)	***
Q-value	1.06 ± 0.01 (15)	1.15 ± 0.01 (19)	***

Data are presented as mean ± SEM; numbers in parenthesis represent number of cells. Cells were recorded at 30 °C. RMP, resting membrane potential; Sag measured at −60 mV; Frequency preference (F_Res_) and resonance strength (Q-value) measured at −70 mV.

*** *p < 0.001, n.s.: p > 0.05, unpaired two-sample Student's t-test*.

The voltage-dependent activation of the conductance(s) responsible for this sag has commonly been associated with resonance properties. Therefore, resonance properties of CA3 and CA1 pyramidal neurons were estimated from a standard ZAP protocol (Hutcheon and Yarom, 2000; Pike et al., 2000). Briefly, a 40 pA peak-to-peak oscillatory current of increasing frequency from 0 to 20 Hz was applied to the cell and the impedance was calculated from the membrane potential response as a function of the frequency (Figures 2A,B). A frequency preference could be determined when the impedance reached a peak in 15 out of 40 CA3 cells and in 19 out of 21 CA1 cells (Table 3). CA3 pyramidal neurons were separated into two groups according to their frequency preference: cells with resonance at a frequency higher than 1 Hz and cells with resonance at lower frequency or not measurable (Figures 2D,E). For most CA1 cells and CA3 cells with resonance above 1 Hz, the frequency preference increased with hyperpolarization of the membrane holding potential (Figures 2C–E). When compared at −70 mV, CA1 pyramidal neurons showed resonance at both a higher frequency (CA3: 1.19 ± 0.09 Hz, *n* = 15; CA1: 2.49 ± 0.17 Hz, *n* = 19; *p* < 10^−4^, unpaired two-sample Student's *t*-test; Table 3) and with greater Q-value (CA3: 1.06 ± 0.01, *n* = 15; CA1: 1.15 ± 0.01, *n* = 19; *p* < 10^−4^, unpaired two-sample Student's *t*-test; Table 3) than CA3 pyramidal neurons.

### 3.2. Bidirectional phase response curve in CA3 and CA1 pyramidal neurons

**Figure 3:**
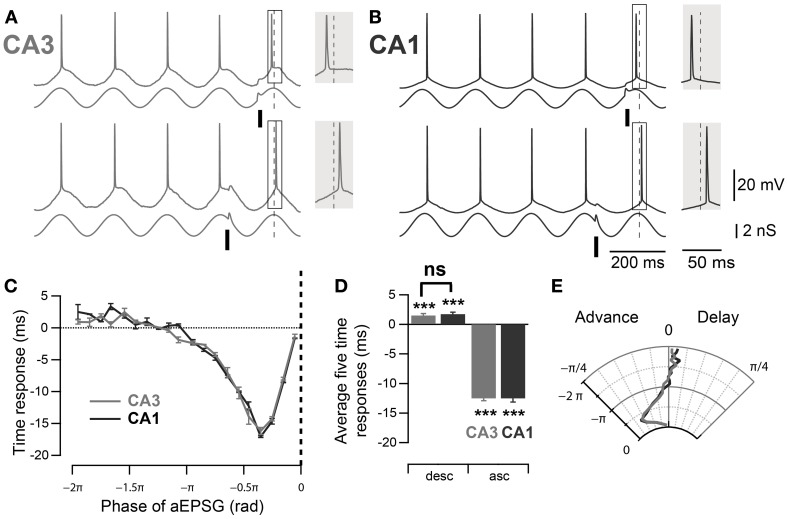
**Comparable PRCs measured at the soma of mouse CA3 and CA1 pyramidal neurons. (A,B)** Representative traces, in CA3 and CA1 respectively, of spike phase shift in response to excitatory input (aEPSG) delivered at different phases of a theta oscillation imposed by dynamic clamp. When aEPSG was delivered at ascending phases, the following spike was advanced compared to the average spike phase over the four preceding cycles (top traces). In contrast, when aEPSG was delivered at descending phases, the following spike was delayed compared to the control spike phase (bottom traces). The spike phase shifts (boxes) are enlarged in the shaded insets. **(C)** Spike time response curve: plot of spike time shift as a function of aEPSG phase relative to the imposed theta oscillation. **(D)** Average spike time response induced by aEPSGs at five descending and ascending phases. Neurons in both CA3 and CA1 showed significant delay after excitatory input at the descending phase of theta oscillation. **(E)** Polar graph of the phase response curve. Data are shown as mean ± SEM, *n* = 32 for CA3 and 11 for CA1. ^***^p < 0.001, one-sample mean angle test; ns: Watson–Williams test; rad, radian; asc, ascending phases; desc, descending phases.

In order to compare the spike timing response between mouse CA3 and CA1 pyramidal neurons, their phase response curves were measured using dynamic clamp. Artificial conductances were applied at the soma of the pyramidal neurons to mimic the inhibitory theta oscillation (3 nS) and excitatory inputs (aEPSGs, 1 nS). The aEPSGs were delivered at different phases of the induced 5 Hz oscillation and their effect on the successive spike phase was measured, compared to a control spike phase averaged from the four immediately preceding cycles. As previously described in rat CA3 (Lengyel et al., 2005), aEPSGs advanced the subsequent spike when delivered at the ascending phase of the theta oscillation (Figures 3A,C–E, ascending phases: −0.39 ± 0.01 rad, *n* = 32, *p* < 10^−4^, one-sample mean angle test) and led to a significant delay of the following spike when delivered at the descending phase (Figures 3A–E; descending phases: 0.04 ± 0.01 rad, *n* = 32, *p* = 10^−4^, one-sample mean angle test). Moreover, as suggested by previous work (Kwag and Paulsen, 2009a), a similar effect was seen in CA1 pyramidal neurons (Figures 3B–E; ascending phases: −0.39 ± 0.02 rad, *n* = 11, *p* < 10^−4^; descending phases: 0.05 ± 0.01 rad, *n* = 11, *p* < 10^−4^; one-sample mean angle test). We then quantitatively compared the phase response curves between CA3 and CA1 (Figures 3C–E). Quantifications were done by averaging spike phase responses for stimulations occurring in the first descending π/2 rad and the last ascending π/2 rad of theta cycles. There was no significant difference in spike phase delay induced by stimulation at descending phases of the theta oscillation between CA3 and CA1 (descending phases CA3 *vs*. CA1: *p* = 0.98, Watson–Williams test); nor was there a difference in spike phase advance in response to aEPSGs delivered at ascending phases (ascending phases CA3 *vs*. CA1: *p* = 1, Watson–Williams test).

### 3.3. Effect of the conductance magnitude of the imposed oscillation

**Figure 4:**
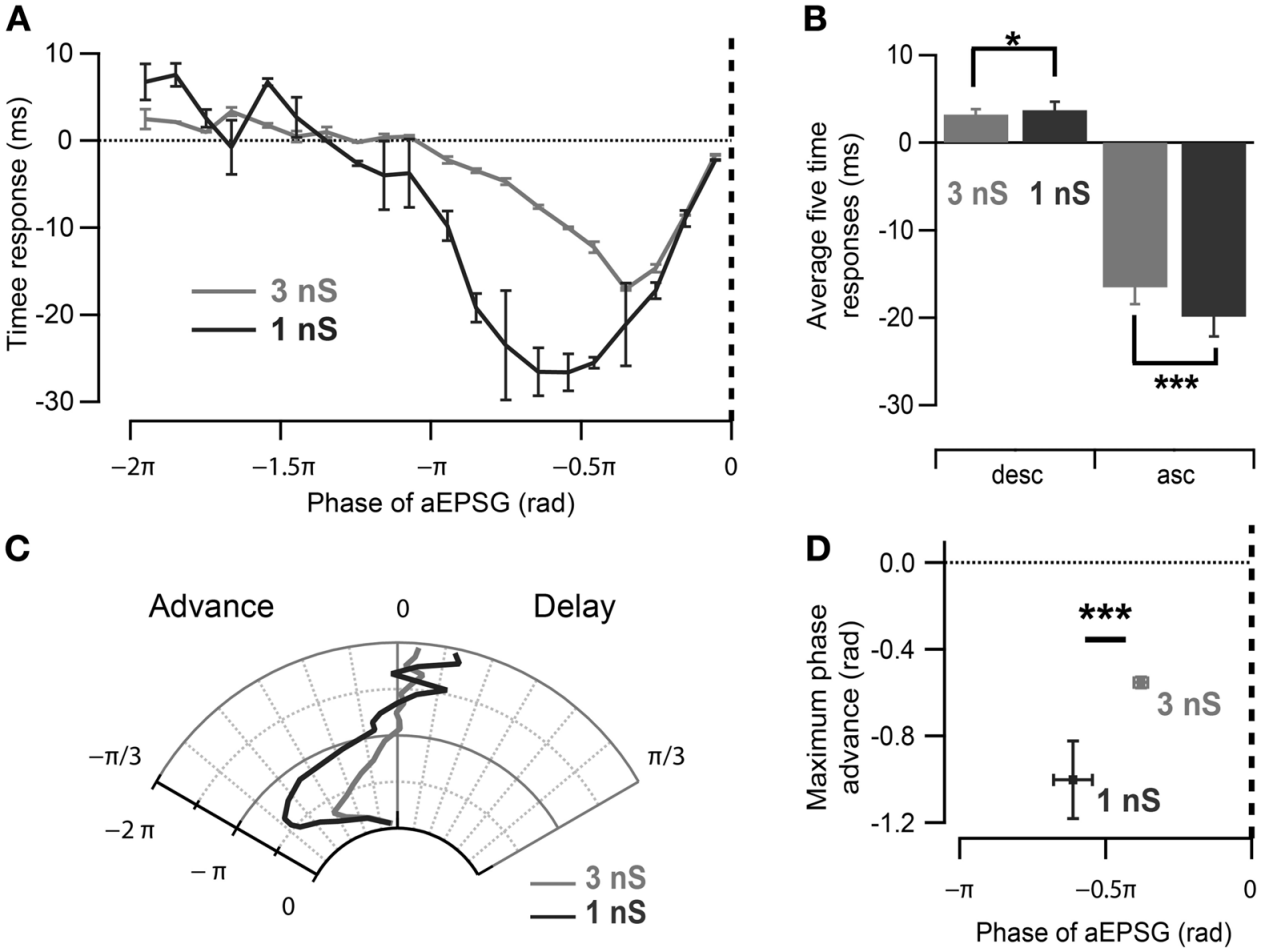
**Effect of oscillation strength on the phase response curve. (A)** Spike time response curve of CA1 pyramidal neurons measured for strong (3 nS) and weak (1 nS) induced oscillation. Note differences in the input phase for maximum spike time advance as well as the magnitude of time advance. **(B)** Spikes have a tendency to be more advanced and delayed by aEPSGs delivered at 0.5π rad of the ascending and descending slope of 1 nS than 3 nS-induced oscillation respectively. ^*^p < 0.05, ^***^p < 0.001, Watson–Williams test. **(C)** Polar representation of the phase response curves in **(A)**. **(D)** Maximal spike phase advance occurs significantly earlier with excitatory inputs during oscillations with smaller maximum conductance in CA1 pyramidal neurons. Data are shown as mean ± SEM, *n* = 11 for 3 nS and *n* = 5 for 1 nS oscillatory inhibition; ^***^p < 0.001, Watson–Williams test; rad, radian; asc, ascending phases; desc, descending phases.

As the neuronal phase response to aEPSGs is likely to reflect the interaction of intrinsic properties and imposed oscillation, we next investigated the effect of the magnitude of the imposed oscillatory conductance in CA1 cells. By decreasing it from 3 to 1 nS (Figure 4A), the average spike phase delay and advancement measured for aEPSGs delivered during π/2 rad of the ascending and descending oscillation, respectively, tended to increase (Figures 4B–D; ascending phases of 1 nS oscillation: −0.63 ± 0.07 rad, *p* = 0.001; descending phases of 1 nS oscillation: 0.12 ± 0.03 rad, *p* = 0.03; *n* = 5, Watson–Williams test). Interestingly, the profile of the phase response curve (PRC) was altered as spikes were advanced in response to aEPSGs delivered at earlier phases of the oscillation (Figures 4A,C; phase of the aEPSGs producing the maximal phase advance: during 3 nS oscillation: −0.38 ± 0.02π rad, *n* = 11; during 1 nS oscillation: −0.61 ± 0.06π rad, *n* = 5; *p* = 0.001, Watson–Williams test).

### 3.4. Role of h-current in spike phase response

**Figure 5:**
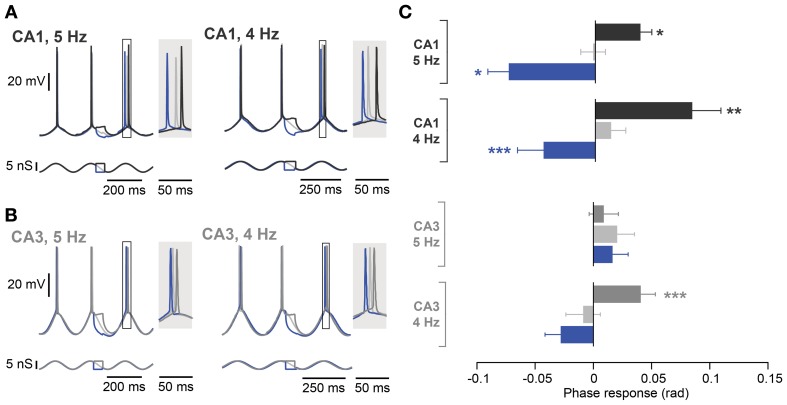
**Frequency dependence of spike phase control recorded at 5 Hz and 4 Hz. (A)** Representative responses of a CA1 neuron (top traces) to inputs optimized for modulating *I*_*h*_ during induced oscillation (bottom traces) at 5 Hz (left panel) and 4 Hz (right panel). The spike phase shifts (boxes) are enlarged in the shaded insets. **(B)** Same as in **(A)** but from a CA3 neuron with a resonance peak > 1 Hz. **(C)** Average phase responses of CA1 and CA3 pyramidal neurons for both a 5 and 4 Hz oscillation. In CA1, a depolarizing step delayed the following spike, whereas a hyperpolarizing step advanced the following spike compared to the control condition at both oscillation frequencies. CA3 neurons, however, even with a resonance peak > 1 Hz, failed to show phase delay and advance with stimulation during descending phases of an ongoing 5 Hz oscillation. The spike phase delay is partially rescued when the frequency of the imposed oscillation is lowered to 4 Hz (*n* = 4). Data are shown as mean ± SEM. ^*^p < 0.05, ^**^p < 0.001, ^***^p < 0.0001, Moore's test; rad, radian.

Given the differences in intrinsic membrane properties between CA3 and CA1 pyramidal neurons (Table 3), it appeared surprising that we were unable to detect any significant difference in phase response properties between the two cell types. We therefore designed a new protocol to maximize possible differences between CA3 and CA1 pyramidal neurons. To this end, the oscillation was driven by dynamic clamp with a 5 nS inhibitory conductance. Moreover, perturbations were optimized for descending phases of the oscillation by increasing their duration to one quarter of an oscillatory cycle (between −1.8π and −1.3π rad). The effect of excitation and inhibition were estimated as the spike phase response to subthreshold depolarizing and hyperpolarizing steps compared to a control condition when no perturbation was added (Figures 5A,B). As expected (Kwag and Paulsen, 2009a), depolarizing steps led to a delay and hyperpolarizing steps led to advancement of the subsequent spike in CA1 during the 5 Hz oscillation (Figure 5C; control: −0.02 ± 0.01 rad; depolarization: 0.04 ± 0.01 rad, *p* = 0.02; hyperpolarization: −0.07 ± 0.02 rad, *p* = 0.02; *n* = 5, Moore's test). This effect was prevented by the *I*_*h*_ blocking drug ZD7288 (10 μM, *n* = 5; control: 0.02 ± 0.01 rad; depolarization: 0.00 ± 0.02 rad, *p* = 0.67; hyperpolarization: 0.09 ± 0.04 rad, *p* = 0.25; *n* = 5, Moore's test; data not shown) and was absent in mice with a genetic deletion of h-channels (HCN1 KO, *n* = 5; control: 0.0 ± 0.01 rad; depolarization: −0.01 ± 0.02 rad, *p* = 0.67; hyperpolarization: 0.04 ± 0.04 rad, *p* = 0.45; *n* = 5, Moore's test; data not shown).

**Figure 6:**
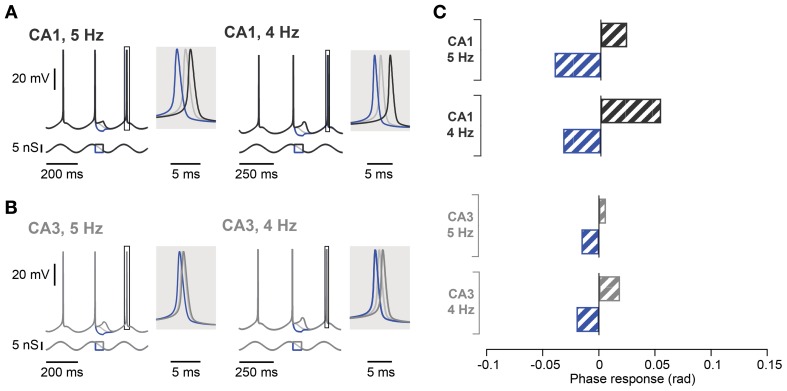
**Spike phase control in computational models of hippocampal CA1 and CA3 pyramidal neurons. (A)** Voltage response of CA1 neuron model (top traces) in response to excitatory and inhibitory perturbation of an oscillatory input (bottom traces) at 5 Hz (left panel) or 4 Hz (right panel). The voltage response around the control spike is expanded to display spike phase responses. The spike phase shifts (boxes) are enlarged in the shaded insets. **(B)** Same as in **(A)** but in CA3 neuron model with h-channel kinetics adjusted to reproduce the voltage sag seen in CA3 neurons. **(C)** Summary spike phase changes elicited in CA1 and CA3 neuron model by excitatory and inhibitory step inputs during 5 and 4 Hz oscillation. rad, radian.

In contrast to the effect in CA1, during a 5 Hz oscillation this protocol failed to induce any significant phase response from CA3 pyramidal neurons, even those selected for their resonance properties (Figure 5C; control: 0.02 ± 0.01 rad; depolarization: 0.01 ± 0.01 rad, *p* = 0.62; hyperpolarization: 0.02 ± 0.01 rad, *p* = 0.99; *n* = 5, Moore's test). Since not only the strength of resonance but also the frequency preference differed between CA3 and CA1 pyramidal neurons (Table 3), we next investigated the influence of the *I*_*h*_ kinetics. To that aim, a computational model of CA3 and CA1 pyramidal neurons was generated in which the main distinction was *I*_*h*_ activation time constants and maximal conductance. *I*_*h*_ kinetics were modified according to experimental data so that CA3 model cells had half the sag in response to a negative step of current compared to the CA1 model (Table 3; Figure 1B). With those parameters, the model demonstrated a more prominent spike phase response during 5 Hz oscillation in CA1 than in CA3 neuron models (Figures 6A–C). The model also suggested that decreasing the oscillatory frequency to 4 Hz would increase the spike phase response in CA3 cells (Figures 6B,C).

Experimentally, we confirmed that the spike phase delay resulting from subthreshold steps during the descending phase of an oscillation was rescued in CA3 cells with resonance peak exceeding 1 Hz by reducing the oscillation frequency to 4 Hz (Figure 5C; control: −0.01 ± 0.01 rad; depolarization: 0.04 ± 0.01 rad, *p* < 10^−4^; hyperpolarization: −0.03 ± 0.01 rad, *p* = 0.35; *n* = 4, Moore's test). A reduction in oscillation frequency had only a minor effect on CA1 cells (Figure 5C; control: 0.01 ± 0.01 rad; depolarization: 0.08 ± 0.02 rad, *p* = 0.001; hyperpolarization: −0.04 ± 0.02 rad, *p* = 0.0004; *n* = 10, Moore's test).

## 4. Discussion

This study revealed a frequency-dependent difference between CA3 and CA1 spike phase responses based on differential properties of the *I*_*h*_ current in these two cell classes. The stronger expression and faster kinetics of *I*_*h*_ in CA1 pyramidal neurons was reflected by a larger voltage sag ratio in response to injection of negative current steps, compared to CA3, together with a more prominent resonance peak than that in CA3 cells. Low conductance oscillations promoted precise temporal spike pattern of CA3 and CA1 pyramidal neurons, bidirectionally controlled by their inputs. The involvement of *I*_*h*_ in the spike phase shift induced by inputs at descending phases of the membrane potential oscillation was confirmed by the impairment of the response when *I*_*h*_ was blocked by a selective blocker or by a genetic knockout of HCN1 channels. Moreover, the active spike phase response was reduced by a strong oscillatory conductance. As predicted by a computational model, and consistent with the kinetics of *I*_*h*_ activation in the two cell types, the active spike phase response was much reduced in CA3 pyramidal neurons at 5 Hz but rescued at a lower frequency. Our results support the view that intrinsic properties of hippocampal cells allow temporal control of their output by the interaction of inputs with active membrane properties during oscillatory network activity.

CA3 and CA1 pyramidal neurons display different membrane properties consistent with differential expression of *I*_*h*_. This conclusion is supported by two independent measurements, namely the sag ratio and the resonance frequency of the two cell types, and is in agreement with previous reports. In CA1 pyramidal neurons, our data are consistent with previous reports concerning the sag ratio (Zemankovics et al., 2010), the frequency preference (Hu et al., 2002) and the magnitude of the resonance (Hu et al., 2002; Zemankovics et al., 2010). In CA3 pyramidal neurons, the more modest sag ratio and resonance properties are consistent with the work of Vasilyev and Barish (2002), measuring a slower and smaller *I*_*h*_ in CA3 compared to CA1 cells. This can explain why only a subpopulation of pyramidal neurons in CA3 had a detectable resonance peak.

Despite different membrane properties, CA3 and CA1 pyramidal neurons have similar spike phase responses. This similarity reflects their comparable passive membrane properties and especially *R*_*in*_. The active component of their response, however, although based on *I*_*h*_ activation, is surprisingly similar despite differences in *I*_*h*_ between the two cell types. This could be accounted for by the experimental design, which drives the membrane potential oscillation and mimics artificial inputs with relatively low conductances using dynamic clamp. This allows the smaller *I*_*h*_ conductance in CA3 neurons to influence spike timing. Bidirectional spike phase responses have previously been reported in both CA3 (Lengyel et al., 2005) and CA1 (Kwag and Paulsen, 2009a) pyramidal neurons. Differences in the magnitude of the delay effects between the studies most likely result from differences in the species used, the strength of the oscillation imposed, and in the analysis procedures, particularly in the handling of directly activated spikes. In view of these results, the place field stability described by Hussaini et al. (2011) in HCN1 KO animals could result from the impairment of spike phase shift responsible for the appropriate experience-dependent changes in synaptic weights.

An interaction between the injected conductance and the intrinsic conductances was seen when the membrane potential oscillation of CA1 pyramidal neurons was driven with a weaker conductance. In this case, the passive and active spike phase responses (based on artificially injected and *I*_*h*_-activated conductances respectively) are more prominent. The larger *I*_*h*_ in CA1 pyramidal neurons allows an active spike phase control even for strong oscillation of the membrane potential. With a more modest expression of *I*_*h*_, in contrast, CA3 pyramidal neurons appear to fail to activate the *I*_*h*_ conductance sufficiently to overcome the injected oscillatory conductance. Interestingly, the gradient expression of *I*_*h*_ along the dendritic tree (Magee, 1998) predicts stronger spike phase control resulting from extracellular stimulation in apical dendritic layers. Inputs would yield different control of spike phase depending on their location and the local *I*_*h*_, although additional synaptic conductances may also contribute (Kwag and Paulsen, 2009b).

The differences in *I*_*h*_ properties between CA3 and CA1 pyramidal neurons involve not only the total conductance but also the activation kinetics, and the spike phase response in CA3 pyramidal neurons was rescued during slower oscillations. This effect was predicted by computational modeling of the two cell types based on known morphological differences (Ishizuka et al., 1995; Morellini et al., 2010) and the recorded intrinsic membrane properties. The model was designed as a single compartment and conformed to our *in vitro* experiments for which current injection, conductance simulation, and membrane potential recordings were done at the soma. It has to be noted, nonetheless, that this model did not explicitly model the spatial recruitment of the dendritic conductances (Káli and Zemankovics, 2012). The activation kinetics for *I*_*h*_, although modified to fit our data, remain close to the source equation originating from Morse et al. (2010). The frequency-dependence of CA3 pyramidal neuron spike phase control could be of functional importance with regards to information processing. The slower oscillation used in this study, namely 4 Hz, is in the lower range of the theta band. Interestingly, a reduction of theta frequency accompanies novelty *in vivo* (Jeewajee et al., 2008). *I*_*h*_ expression in CA3 pyramidal neurons might therefore account for a separation between encoding and retrieval states in this subfield and internal associational connections storing previous experiences could then be strengthened before being reconnected to primary sensory information in CA1.

### Conflict of interest statement

The authors declare that the research was conducted in the absence of any commercial or financial relationships that could be construed as a potential conflict of interest.
